# Supplementary material for: Rapid stimulus-driven modulation of slow ocular position drifts
Source: eLife. 2020 Aug 6;9:e57595. doi: 10.7554/eLife.57595 (PMC7442486; doi:10.7554/eLife.57595)
Supplement: Transparent reporting form [file elife-57595-transrepform.docx]

***eLife’s* transparent reporting form**

We encourage authors to provide detailed information *within their submission* to facilitate the interpretation and replication of experiments. Authors can upload supporting documentation to indicate the use of appropriate reporting guidelines for health-related research (see [EQUATOR Network](http://www.equator-network.org/%20)), life science research (see the [BioSharing Information Resource](https://biosharing.org/)), or the [ARRIVE guidelines](http://www.plosbiology.org/article/info:doi/10.1371/journal.pbio.1000412) for reporting work involving animal research. Where applicable, authors should refer to any relevant reporting standards documents in this form.

If you have any questions, please consult our Journal Policies and/or contact us: [editorial@elifesciences.org](mailto:editorial@elifesciences.org).

**Sample-size estimation**

- You should state whether an appropriate sample size was computed when the study was being designed
- You should state the statistical method of sample size computation and any required assumptions
- If no explicit power analysis was used, you should describe how you decided what sample (replicate) size (number) to use

Please outline where this information can be found within the submission (e.g., sections or figure legends), or explain why this information doesn’t apply to your submission:

We did not explicitly estimate sample size. However, repeating the experiments on 3 different monkeys, with consistent results throughout all of them, is beyond the current minimum standard of 2 monkeys per study (due to compromises associated with the 3R principle for the use of non-human primates in research). In our figures, we reported each individual monkey’s data separately, to highlight the consistency of the results across animals. We also typically had hundreds, if not thousands, of trial repetitions per shown condition. This increases our confidence in our results. In only one panel of one figure (Fig. 4G), we did not report averages because there were fewer than 10 repetitions in them (due to the specific biological constraint associated with this analysis of rare microsaccades shortly after stimulus onset).

**Replicates**

- You should report how often each experiment was performed
- You should include a definition of biological versus technical replication
- The data obtained should be provided and sufficient information should be provided to indicate the number of independent biological and/or technical replicates
- If you encountered any outliers, you should describe how these were handled
- Criteria for exclusion/inclusion of data should be clearly stated
- High-throughput sequence data should be uploaded before submission, with a private link for reviewers provided (these are available from both GEO and ArrayExpress)

Please outline where this information can be found within the submission (e.g., sections or figure legends), or explain why this information doesn’t apply to your submission:

Trial repetitions for the experiments are reported in Methods under the section “Behavioral tasks” and also in the appropriate figures.

We randomly interleaved the relevant conditions for statistical comparisons (e.g. control versus full-screen flash) in equal proportions, as described in Methods.

The “Data analyses” section of methods describes exclusion and inclusion criteria of data for specific analyses. For example, we removed trials with blinks. For some analyses, we removed trials with saccades, but for others, we kept them, depending on the specific question being investigated.

The only outlier scenario that we encountered was associated with small numbers of observations in a specific condition (due to the biological phenomenon of saccadic inhibition). In this case, we excluded from analysis the conditions that had fewer than 10 observations (Fig. 4G).

**Statistical reporting**

- Statistical analysis methods should be described and justified
- Raw data should be presented in figures whenever informative to do so (typically when N per group is less than 10)
- For each experiment, you should identify the statistical tests used, exact values of N, definitions of center, methods of multiple test correction, and dispersion and precision measures (e.g., mean, median, SD, SEM, confidence intervals; and, for the major substantive results, a measure of effect size (e.g., Pearson's r, Cohen's d)
- Report exact p-values wherever possible alongside the summary statistics and 95% confidence intervals. These should be reported for all key questions and not only when the p-value is less than 0.05.

Please outline where this information can be found within the submission (e.g., sections or figure legends), or explain why this information doesn’t apply to your submission:

For each monkey, we showed examples of raw eye movement data (e.g. Fig. 1, Fig. 1-figure supplement 1C, I). We also showed raw microsaccade times (e.g. Fig. 4, Fig. 4-figure supplement 1). In fact, the bulk of our analyses are not far removed from the raw traces of eye position that we collected, with only minimal analyses (like computing averages across repetitions).

Statistical analysis methods are also described in a dedicated “Statistics” section in Methods.

Exact N values and p-values are reported in the Results text, and in the figures and figure legends. Multiple correction methods are defined in the “Statistics” section of Methods. Moreover, measures of central tendency and dispersion are all defined in the figure legends. Confidence intervals are also shown in the figures, and defined in the figures and legends.

(For large datasets, or papers with a very large number of statistical tests, you may upload a single table file with tests, Ns, etc., with reference to sections in the manuscript.)

**Group allocation**

- Indicate how samples were allocated into experimental groups (in the case of clinical studies, please specify allocation to treatment method); if randomization was used, please also state if restricted randomization was applied
- Indicate if masking was used during group allocation, data collection and/or data analysis

Please outline where this information can be found within the submission (e.g., sections or figure legends), or explain why this information doesn’t apply to your submission:

No group allocation was needed in this study.

**Additional data files (“source data”)**

- We encourage you to upload relevant additional data files, such as numerical data that are represented as a graph in a figure, or as a summary table
- Where provided, these should be in the most useful format, and they can be uploaded as “Source data” files linked to a main figure or table
- Include model definition files including the full list of parameters used
- Include code used for data analysis (e.g., R, MatLab)
- Avoid stating that data files are “available upon request”

Please indicate the figures or tables for which source data files have been provided:

We practically show raw data in all of our figures. Our measurements of eye position are also standard in the field. We can upload the data associated with figures upon acceptance.
